# Association between serum α1-antitrypsin levels and all-cause mortality in the general population: the Nagahama study

**DOI:** 10.1038/s41598-021-96833-3

**Published:** 2021-08-26

**Authors:** Yasuharu Tabara, Kazuya Setoh, Takahisa Kawaguchi, Shinji Kosugi, Takeo Nakayama, Fumihiko Matsuda

**Affiliations:** 1 Graduate School of Public Health, Shizuoka Graduate University of Public Health, Aoi-ku, Shizuoka, 420-0881 Japan; 2 Center for Gnomic Medicine, Kyoto University Graduate School of Medicine, Sakyo-ku, Kyoto, 606-8507 Japan; 3 Department of Medical Ethics and Medical Genetics, Kyoto University School of Public Health, Kyoto, Japan; 4 Department of Health Informatics, Kyoto University School of Public Health, Sakyo-ku, Kyoto, 606-8507 Japan

**Keywords:** Risk factors, Public health

## Abstract

Circulating levels of inflammatory proteins have to be prognostic markers of all-cause mortality. α1-Antitrypsin (AAT) is a major inflammatory plasma protein, but its association with all-cause mortality is unclear. We aimed to evaluate the prognostic significance of AAT levels for all-cause mortality. Study participants comprised 9682 community residents (53.5 ± 13.3 years old). During the 9.8-year follow-up period, 313 participants died from any cause. The mortality rate increased linearly with AAT quintiles (Q1, 18.2; Q2, 24.7; Q3, 23.8; Q4, 31.9; Q5, 64.6 per 10,000 person-years). There were significant correlations between AAT and high-sensitivity C-reactive protein (hsCRP) levels (correlation coefficient, 0.331; *P* < 0.001). However, the Cox model analysis, when adjusted for possible covariates including hsCRP, identified the fifth AAT quintile as a risk factor for all-cause death (hazard ratio, 2.12 [95% confidence interval, 1.41–3.18]; *P* < 0.001). An analysis of participants older than 50 years (hazard ratio, 1.98, *P* < 0.001) yielded similar results. The hazard ratio increased proportionately in combination with high AAT and high hsCRP levels, and the highest hazard ratio reached 4.51 (95% confidence interval, 3.14–6.54, *P* < 0.001). High AAT levels were determined to be an independent risk factor for mortality in the general population.

## Introduction

Circulating levels of inflammatory markers, particularly C-reactive protein (CRP), are reportedly associated with cardiovascular diseases (CVDs) morbidity, as well as CVD and all-cause mortality^1,2^. However, CRP alone may not be discriminative enough to assess the risk of outcomes. Therefore, the recommendations from The United States Preventive Services Task Force^3^ and the 2016 European Guidelines on cardiovascular disease prevention^4^ do not support CRP monitoring of asymptomatic adults due to the slight added benefit of modifying risk reclassification by adding CRP to the existing CVD risk assessment models, though the CRP assessment may be used as part of refined risk assessment in patients with moderate CVD risk profile^4^. Recently, several studies^5–7^ reported a strong relationship between glycoprotein acetyl (GlycA) signals, as measured by proton nuclear magnetic resonance, and CVD events. Because the GlycA signal is a composite measure of N-glycosylated proteins, including acute-phase reactant proteins such as α1-acid glycoprotein, α1-antitrypsin (AAT), α1-antichymotrypsin, haptoglobin, and transferrin^8–10^, the combination of CRP with these low-grade inflammatory markers may increase its evaluative capacity for the risk of CVD and mortality. Among them, AAT, a major serine protease inhibitor, is an abundant protein that was reported to be independently associated with CVD in general population-based studies^11–15^; i.e., elevated AAT levels^10^, as well as chronic inflammation assessed by six inflammatory markers including AAT^12–15^, were associated with coronary heart diseases^11,12,14,15^, stroke^11,13,14^, and cardiovascular^12,14,15^ and all-cause mortality^12,14^. Although these studies were based on the same male population, the results indicated that circulating AAT levels can serve as an inflammatory marker of the potential risk associated with future cardiovascular events and mortality.

Given this background, we investigated associations between circulating AAT levels, as well as its combination with high-sensitivity CRP (hsCRP), and all-cause mortality in a large-scale general population with a follow-up period of approximately 10 years.

## Results

**Figure 1:**
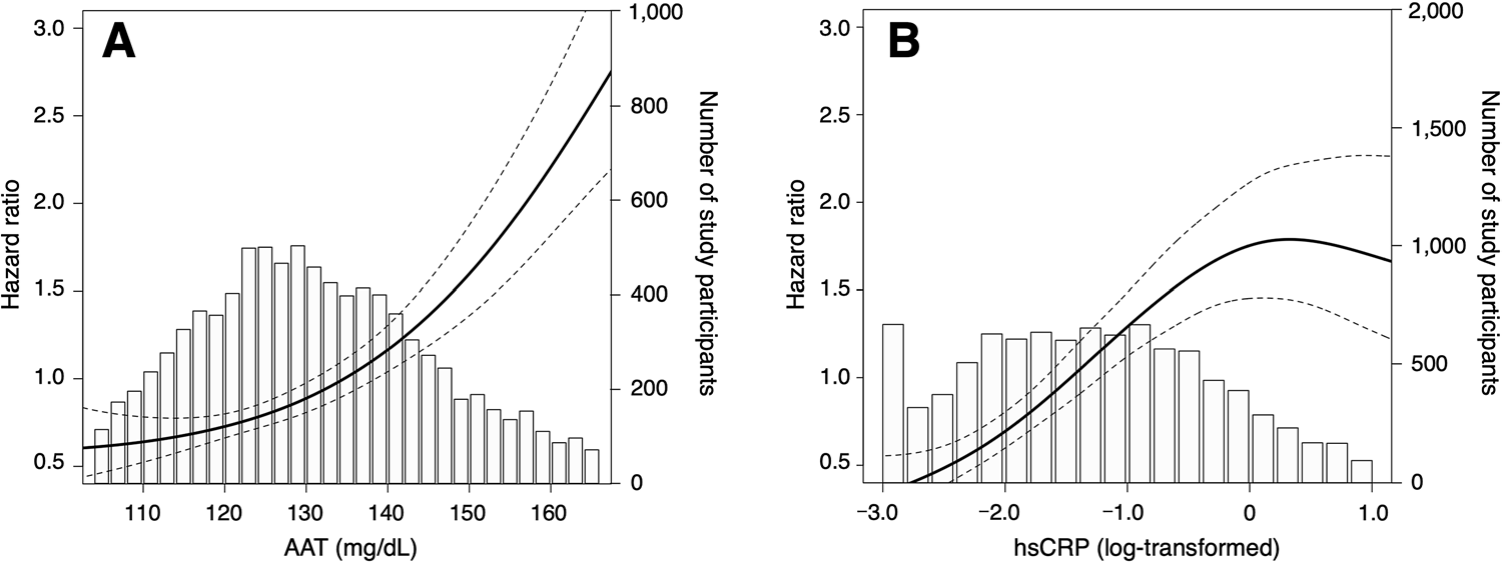
Penalized cubic splines of the association between baseline AAT and hsCRP levels and the hazard ratio for all-cause mortality. Lines indicate the hazard ratio and 95% confidence interval (CI) of baseline. (**A**) α1-Antitrypsin (AAT) and (**B**) log-transformed high-sensitive C-reactive protein (hsCRP) for all-cause mortality. Bar graph indicates number of study participants.

The mean age of the study participants was 53.5 ± 13.3 years, and 32.6% were men. The Spearman's rank correlation coefficient between AAT (mean ± standard deviation: 132 ± 19 mg/dL) and hsCRP (0.90 ± 3.55 μg/mL) was 0.252 (*P* < 0.001). The intra- and inter-assay coefficients of the variation in AAT levels were 1.07–1.67% and 1.02–1.31%, respectively. During the follow-up period (mean follow-up duration, 9.8 years), 313 participants died from any cause. Figure 1 illustrates cubic splines depicting the association between baseline AAT or hsCRP and the hazard ratio (HR) for all-cause mortality.

**Figure 2:**
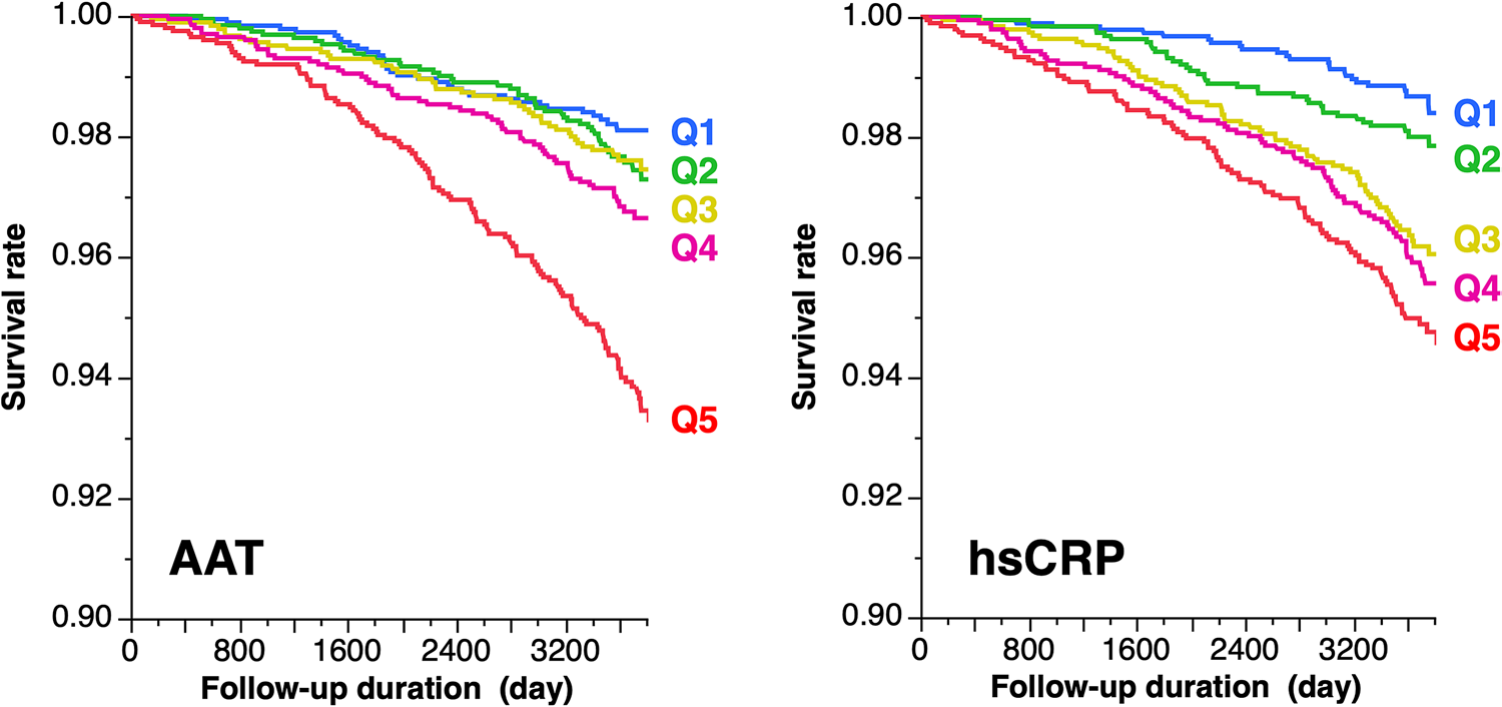
Survival curve of all-cause mortality by the quintiles of baseline α1-antitrypsin (AAT) and high-sensitivity C-reactive protein (hsCRP) levels.

Baseline clinical characteristics of the study participants for the AAT quintiles are shown in Table 1. hsCRP levels increased proportionally with the AAT quintiles, while other clinical traits listed in Table 1 showed no clear relationship with the quintiles. In contrast, almost all clinical traits, including AAT, exhibited positive or inverse relationships with the hsCRP quintiles in a stepwise manner (Table 2). Figure 2 depicts the Kaplan–Meier curves for all-cause death according to the AAT and hsCRP quintiles (log-rank test, all *P* < 0.001). Table 3 provides the summary statistics of the mortality analysis. The Cox proportional hazard analysis adjusted for possible covariates indicated the highest AAT quintile as an independent risk factor for all-cause death in the analysis of the total study population (Table 4). The analysis, which excluded participants who died within 360 days after baseline investigations (N = 8), produced similar results (AAT Q5, HR = 2.11 (1.40–3.16), *P* < 0.001). Furthermore, the prognostic significance of AAT remained significant when AAT was included in the Cox model as a continuous variable (per 10 mg/dL, HR = 1.15 (1.10–1.22), *P* < 0.001). Given the minimum value of AAT in this population (52 mg/dL), none of the participants exhibited severe AAT deficiency, e.g., homozygote of the Glu342Lys disfunction allele of the *SERPINA1* gene shows serum AAT levels less than 45 mg/dL^16^. When excluding suspected cases of AAT deficiency (< 100 mg/dL, N = 204) from the analysis, the HR of AAT did not change substantially (per 10 mg/dL HR = 1.16 (1.10–1.22), *P* < 0.001).

**Table 1:** Baseline clinical characteristics of study participants by AAT level quintile (N = 9682).

	Q1	Q2	Q3	Q4	Q5	P
	< 117 mg/dL	≥ 117 mg/dL	≥ 126 mg/dL	≥ 134 mg/dL	≥ 145 mg/dL	
	(N = 1866)	(N = 1952)	(N = 1848)	(N = 2014)	(N = 2002)	
Age (in years)	52.5 ± 12.5	53.0 ± 13.1	53.4 ± 13.2	53.7 ± 13.7	55.1 ± 14.0	< 0.001
Sex, men %	40.1	31.5	29.2	29.2	33.4	< 0.001
BMI, kg/m^2^	22.7 ± 3.0	22.3 ± 3.1	22.2 ± 3.2	22.2 ± 3.5	22.1 ± 3.5	< 0.001
Current smoking, %	11.6	12.1	12.7	15.7	19.5	< 0.001
Heavy drinking, %	14.3	8.5	8.6	6.9	7.1	< 0.001
History of cancer, %	3.4	3.0	3.4	2.9	4.0	0.362
History of CVD, %	1.8	2.4	2.3	2.8	4.2	< 0.001
Systolic BP, mmHg	123 ± 17	122 ± 17	123 ± 18	124 ± 18	125 ± 18	< 0.001
Mean BP, mmHg	92 ± 13	91 ± 12	92 ± 13	92 ± 13	92 ± 13	0.004
Diastolic BP, mmHg	77 ± 11	75 ± 11	76 ± 11	76 ± 11	76 ± 11	0.006
HbA1c, %	5.4 ± 0.5	5.5 ± 0.5	5.5 ± 0.6	5.5 ± 0.6	5.5 ± 0.5	0.069
HDL cholesterol, mg/dL	66 ± 17	66 ± 17	66 ± 17	65 ± 17	63 ± 17	< 0.001
LDL cholesterol, mg/dL	124 ± 31	124 ± 31	124 ± 31	123 ± 31	121 ± 31	0.031
Albumin, g/dL	4.5 ± 0.2	4.5 ± 0.2	4.5 ± 0.2	4.5 ± 0.2	4.4 ± 0.2	< 0.001
Creatinine, mg/dL	0.7 [0.6–0.8]	0.7 [0.6–0.8]	0.7 [0.6–0.8]	0.7 [0.6–0.8]	0.7 [0.6–0.8]	< 0.001
ALT, IU/L	19 [15–26]	18 [14–24]	17 [13–24]	17 [13–24]	17 [13–23]	< 0.001
γGTP, IU/L	23 [16–40]	20 [15–33]	20 [15–33]	20 [15–31]	21 [15–34]	< 0.001
hsCRP, μg/mL	0.22 [0.11–0.43]	0.23 [0.12–0.46]	0.26 [0.13–0.57]	0.33 [0.14–0.69]	0.52 [0.20–1.36]	< 0.001

Values are provided as the mean ± standard deviation, median [interquartile range], or frequency. Numbers of study participants in each quintile are shown in parentheses. Statistical significance was assessed by analysis of variance, Kruskal–Wallis test, or a Chi-squared test. Cardiovascular disease (CVD) includes symptomatic stroke, angina pectoris, and myocardial infarction. *AAT* α1-antitrypsin, *BMI* body mass index, *BP* blood pressure, *HbA1c* hemoglobin A1c, *HDL* high-density lipoprotein, *LDL* low-density lipoprotein, *ALT* alanine aminotransferase, *γGTP* gamma-glutamyltransferase, *hsCRP* high-sensitivity C-reactive protein.

**Table 2:** Baseline clinical characteristics of the study participants by high-sensitivity hsCRP level quintile (N = 9682).

	Q1	Q2	Q3	Q4	Q5	P
	< 0.11 μg/mL	≥ 0.11 μg/mL	≥ 0.21 μg/mL	≥ 0.39 μg/mL	≥ 0.77 μg/mL	
	(N = 1918)	(N = 1937)	(N = 1937)	(N = 1944)	(N = 1946)	
Age, years old	47.5 ± 12.7	52.3 ± 13.2	55.1 ± 12.8	56.3 ± 12.7	56.5 ± 13.2	< 0.001
Sex, men %	18.9	27.6	34.3	38.1	44.0	< 0.001
BMI, kg/m^2^	20.3 ± 2.4	21.5 ± 2.7	22.4 ± 2.9	23.4 ± 3.2	23.9 ± 3.8	< 0.001
Current smoking, %	12.4	11.6	14.3	14.8	19.0	< 0.001
Heavy drinking, %	7.4	8.8	8.8	9.7	10.2	0.030
History of cancer, %	2.4	3.3	3.5	3.7	3.9	0.110
History of CVD, %	1.3	2.0	2.5	3.6	4.1	< 0.001
Systolic BP, mmHg	116 ± 15	121 ± 17	125 ± 17	126 ± 17	129 ± 18	< 0.001
Mean BP, mmHg	86 ± 11	90 ± 12	93 ± 13	94 ± 12	95 ± 13	< 0.001
Diastolic BP, mmHg	72 ± 10	75 ± 11	77 ± 11	77 ± 11	79 ± 12	< 0.001
HbA1c, %	5.3 ± 0.4	5.4 ± 0.4	5.4 ± 0.4	5.5 ± 0.6	5.6 ± 0.8	< 0.001
HDL cholesterol, mg/dL	73 ± 16	69 ± 17	65 ± 17	61 ± 16	58 ± 15	< 0.001
LDL cholesterol, mg/dL	114 ± 30	121 ± 30	125 ± 30	127 ± 31	127 ± 32	< 0.001
Albumin, g/dL	4.5 ± 0.2	4.5 ± 0.2	4.5 ± 0.2	4.5 ± 0.2	4.4 ± 0.2	< 0.001
Creatinine, mg/dL	0.6 [0.6–0.7]	0.7 [0.6–0.8]	0.7 [0.6–0.8]	0.7 [0.6–0.8]	0.7 [0.6–0.8]	< 0.001
ALT, IU/L	15 [12–19]	17 [13–22]	18 [14–24]	20 [15–27]	20 [15–29]	< 0.001
γGTP, IU/L	16 [13–22]	19 [14–27]	21 [15–34]	24 [17–40]	29 [19–48]	< 0.001
AAT, mg/dL	127 ± 16	128 ± 16	129 ± 17	132 ± 18	142 ± 22	< 0.001

Values are represented as the mean ± standard deviation, median [interquartile range], or frequency. Numbers of study participants in each quintile are shown in parentheses. Statistical significance was assessed by analysis of variance, Kruskal–Wallis test, or a Chi-squared test. Cardiovascular diseases (CVDs) include symptomatic stroke, angina pectoris, and myocardial infarction. *AAT* α1-antitrypsin, *BMI* body mass index, *BP* blood pressure, *HbA1c* hemoglobin A1c, *HDL* high-density lipoprotein, *LDL* low-density lipoprotein, *ALT* alanine aminotransferase, *γGTP* gamma-glutamyltransferase, *hsCRP* high-sensitivity C-reactive protein.

**Table 3:** All-cause mortality rate by baseline AAT and hsCRP quintiles.

	Q1	Q2	Q3	Q4	Q5
**Baseline AAT quintiles**					
Person-years	18,091	19,065	18,094	19,729	19,663
Number of deaths	33	47	43	63	127
Mortality rate	18.2	24.7	23.8	31.9	64.6
**Baseline hsCRP quintiles**					
Person-years	18,663	18,882	19,022	19,058	19,018
Number of deaths	25	38	72	78	100
Mortality rate	13.4	20.1	37.9	40.9	52.6

The mortality rate is shown per 10,000 person-years. *AAT* α1-antitrypsin, *hsCRP* high-sensitivity C-reactive protein.

**Table 4:** Cox proportional hazard analysis for all-cause mortality.

	Total (N = 9682)			≥ 50 years (N = 5975)		
	HR	95% CI	P	HR	95% CI	P
**hsCRP quintiles**						
Q1		Reference			Reference	
Q2	1.02	0.61–1.70	0.936	1.40	0.79–2.49	0.251
Q3	1.54	0.97–2.46	0.069	1.90	1.10–3.27	0.020
Q4	1.44	0.90–2.32	0.128	1.81	1.05–3.13	0.032
Q5	1.36	0.84–2.21	0.211	1.77	1.02–3.08	0.043
**AAT quintiles**						
Q1		Reference			Reference	
Q2	1.22	0.78–1.91	0.382	1.01	0.63–1.62	0.964
Q3	1.11	0.70–1.75	0.660	1.02	0.64–1.63	0.937
Q4	1.31	0.85–2.02	0.214	1.23	0.79–1.91	0.354
Q5	2.12	1.41–3.18	< 0.001	1.98	1.31–2.98	0.001

Adjusted factors were age, sex, body mass index, current smoking, heavy drinking, history of cancer, history of cardiovascular disease, mean blood pressure, hemoglobin A1c, high-density lipoprotein cholesterol, low-density lipoprotein cholesterol, creatinine, alanine aminotransferase, and gamma-glutamyltransferase. The quintiles of high-sensitivity CRP (hsCRP) and α1-antitrypsin (AAT) levels were calculated for all participants. All independent variables showed *P*-values greater than 0.05 using the Schoenfeld residuals test.

**Figure 3:**
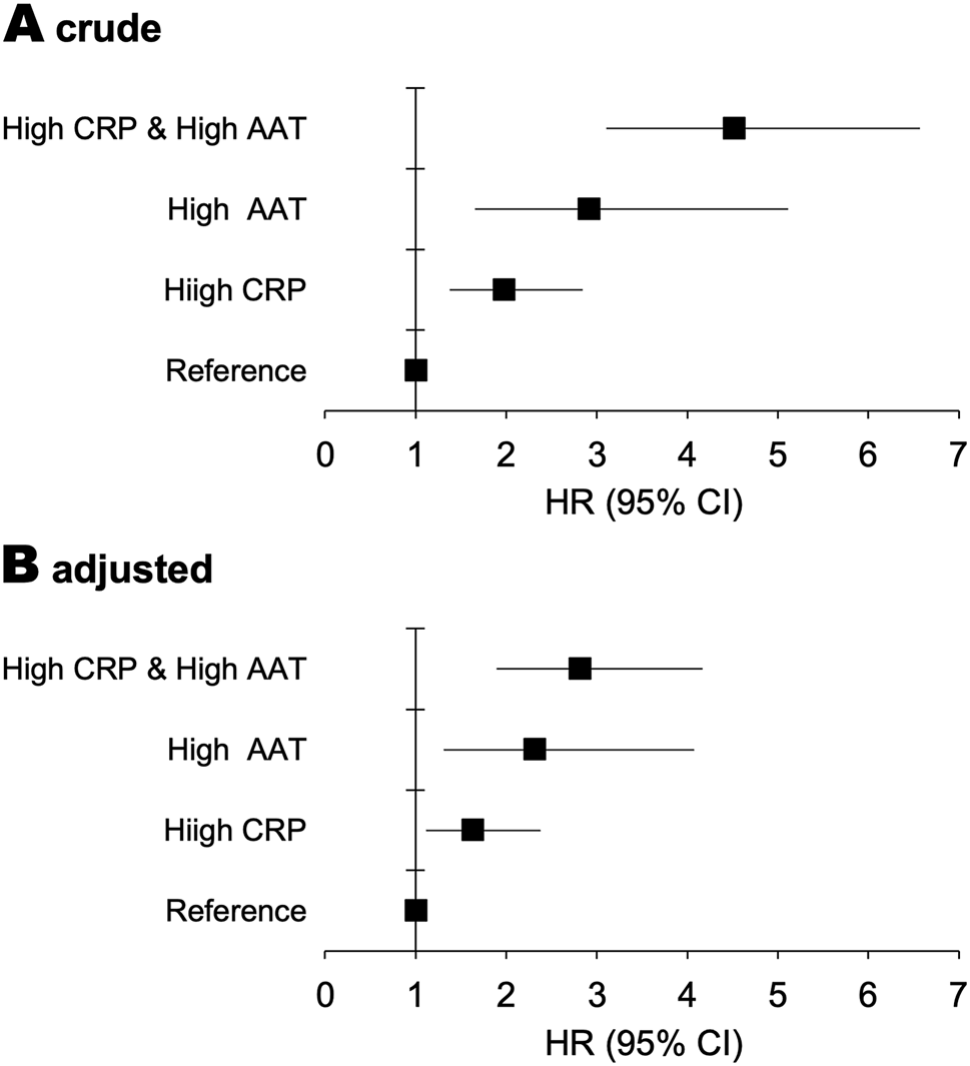
The hazard ratio for all-cause mortality in participants aged ≥ 50 years (N = 5972). High AAT and high CRP were ≥ 145 mg/dL (5th quintile) and ≥ 0.21 μg/mL (3rd to 5th quintiles), respectively. (**A**) Crude hazard ratio (HR) and 95% confidence interval (CI), (**B**) HR adjusted for age, sex, body mass index, current smoking, heavy drinking, history of cancer, history of cardiovascular disease, mean blood pressure, hemoglobin A1c, high-density lipoprotein cholesterol, low-density lipoprotein cholesterol, creatinine, alanine aminotransferase, and gamma-glutamyltransferase. *AAT* α1-antitrypsin, *hsCRP* high-sensitivity C-reactive protein.

An analysis of the sub-population of middle-aged to older (≥ 50 years or older) participants indicated elevated hsCRP (higher than the third quintile) as an independent determinant of all-cause mortality (Table 4), although no significant association was observed in the analysis of all participants. Therefore, the HR increased with the combination of high AAT (5th quintile) and elevated CRP (3rd to 5th quintiles) in the sub-population of older participants (Fig. 3); and the highest HRs observed in the group of both high AAT and elevated CRP were 4.51 (95% CI 3.13–6.65) and 2.81 (95% CI 1.89–4.17) in the crude and covariate-adjusted analyses, respectively.

## Discussion

In this longitudinal study of a large-scale general population, we identified elevated AAT levels as an independent determinant of all-cause mortality. Because this association was independent of hsCRP, the combination of AAT and hsCRP showed a stronger association with all-cause mortality than that of either AAT or hsCRP alone.

Previous epidemiological studies identified systemic inflammatory markers, mainly circulating levels of hsCRP, as independent risk factors for cardiovascular events^1^ and mortality^1,2^; however, the results regarding the prognostic significance of AAT were obtained based on the same population used for the Malmö preventive project^6–11^. Therefore, this is the first study to demonstrate the positive association between AAT and all-cause death in another community dwelling. Reproducible results from a Japanese community of participants of different lifestyle backgrounds would add further evidence to support AAT’s prognostic significance. Although there were significant correlations between AAT and hsCRP levels, the association between AAT and all-cause mortality was more apparent than that between hsCRP and all-cause mortality. In contrast with hsCRP, which was correlated with several clinical risk factors listed in Table 2, as was observed in this study, there was no clear relationship between AAT quintiles and the same clinical risk factors. Although the reasons for the lack of an association between AAT and any of the known risk factors remain uncertain, the independency from other risk factors might be a reason for its more evident association with all-cause mortality than that of hsCRP.

The liver synthesizes most AAT in response to systemic inflammation^17^. Therefore, patients with severe hepatic disease have lower AAT levels^18^. However, the association between high AAT levels and all-cause death was not due to potential hepatic disease or dysfunction; indeed, there were only weak associations between the AAT quintiles and liver enzyme activities. AAT levels are elevated in patients with cancer^19^, but the association between AAT with all-cause mortality was also unrelated to cancer history. Additionally, our longitudinal analysis results did not change substantially when the participants who died within 1 year after baseline investigations were excluded from the analysis, nor did the sensitivity analysis when potential cases of AAT deficiency were excluded. Therefore, the association between AAT and all-cause death might not be due to reverse causation bias due to including participants with serious clinical complications at baseline.

We previously identified the rs671 genotype of the aldehyde dehydrogenase 2 gene as a susceptible locus for circulating AAT levels via genome-wide association analysis of the Nagahama population^20^. Most East Asians, including Japanese people, possess the inactive allele of the gene; the approximate inactive allele frequency is 0.24 among Japanese, according to the HapMap Japanese dataset. Because aldehyde dehydrogenase 2 is a rate-controlling enzyme in ethanol metabolism, those who possess homozygosity for the inactive allele tend to be non-drinkers, while in those who do not possess homozygosity, the daily alcohol consumption of enzymatically active allele homozygotes is reported to be approximately double than that of those who exhibit heterozygosity^21–23^. Although the mechanisms by which this genotype is associated with the AAT level are unclear, the association between AAT and all-cause death observed in this study was independent of a heavy drinking habit, which is a well-established risk factor of mortality in the general population^24^.

By using genotypes that are robustly associated with circulating levels of CRP as an instrumental variable, several Mendelian randomization studies clarified that elevated CRP is a marker, but not a cause, of metabolic syndrome^25^ and coronary events^26^. AAT levels may also reflect potential physiological frailty, thus exhibiting a positive association with all-cause death. Other inflammation-sensitive proteins, such as fibrinogen, have been reported to be associated with cardiovascular outcomes^27^ and death due to cardiovascular events^28^. Although we do not have data on other inflammatory markers, the independent association of AAT and hsCRP supports the possibility that in combination with other inflammatory markers, each would show a stronger association with all-cause mortality. It should be clarified whether GlycA signals, a composite measure of acute-phase reactant proteins, or a specific combination of inflammatory markers show stronger associations with all-cause mortality.

Several study limitations exist. First, we could not investigate the association between AAT and cardiovascular events due to an insufficient number of event cases. Similarly, we could not investigate the potential association with cause-specific death. Further long-term follow-up is needed to elucidate these unknowns.

In conclusion, high AAT was an independent risk factor for all-cause mortality in the general Japanese population. Our results indicate the importance of evaluating AAT levels as a risk factor for adverse outcomes.

## Methods

### Study participants

We conducted a longitudinal analysis utilizing the dataset from the Nagahama study^29,30^, which is an ongoing longitudinal study based on the clinical data of community residents of Nagahama city, Japan. The study participants were recruited between 2008 and 2010 from community residents aged 30–74 years old, who were living independently without physical impairment or dysfunction. Among the baseline population (N = 9764), 9682 participants were included in the study after the exclusion of individuals who met the following exclusion criteria: widely deviating clinical values of gamma-glutamyltransferase (γGTP) (≥ 500 IU/L, N = 15) or creatinine (≥ 2 mg/dL, N = 8), and incomplete data on the clinical values required for this study (N = 59).

All study procedures were approved by the ethics committee of Kyoto University Graduate School of Medicine and by the Nagahama Municipal Review Board. Written informed consent was obtained from all participants. All study procedures were performed in accordance with relevant regulations and guidelines including the Ethical Guidelines for Medical and Health Research Involving Human Subjects in Japan and the declaration of Helsinki.

### Baseline clinical characteristics

The baseline clinical characteristics of the study participants were obtained at the time of recruitment. Serum levels of AAT and hsCRP were measured using a blood sample drawn at the baseline investigations in a for-profit laboratory (SRL Inc., Tokyo, Japan) using the N-antiserum to Human Alpha-1-Antitrypsin Kit or N-Latex CRP II Kit (Siemens Healthcare Diagnostics, Munich, Germany). Other blood markers were measured using the same sera in another commercial laboratory (Medic Inc., Shiga, Japan). The following measurement kits were used for the assessment: albumin, Albumin II-HA test Wako (Fujifilm Wako Co., Ltd., Osaka, Japan); hemoglobin A1c, Determiner L HbA1c (Hitachi Chemical Diagnostics Systems Co., Ltd., Tokyo, Japan); high-density lipoprotein cholesterol, Determiner L HDL-C (Hitachi Chemical); low-density lipoprotein cholesterol, Metaboread LDL-C (Hitachi Chemical); creatinine, Espa CRE-liquid II (Nipro Co., Ltd., Osaka, Japan); alanine aminotransferase, L-type Wako ALT-J2 (Fujifilm Wako); gamma-glutamyl transferase, L-type Wako γ-GT-J (Fujifilm Wako).

Clinical histories and smoking and drinking habits were queried using a structured, self-administered questionnaire. Heavy drinking was defined as ≥ 2 (men) or ≥ 1 (women) Go drink per sitting, where one Go, which is a Japanese traditional liquor unit, corresponds to 22 g ethanol. Brachial blood pressure (BP) was measured twice using a cuff-oscillometric device (HEM-9000AI; Omron Healthcare, Kyoto, Japan), which was performed a few minutes after the test participant had rested in the sitting position. The mean of the two readings was used as the representative value.

### Follow-up and death ascertainment

The primary outcome was all-cause mortality. We followed cases involving relocation out of Nagahama city and those of all-cause death by reviewing residential registry records managed by the Nagahama City Office. We calculated the follow-up period as the duration between baseline investigations and the date of relocation or death, or the current end of the follow-up period (March 31, 2020). Because the first case of SARS-CoV-2 infection in Nagahama city was reported on April 3, 2020, which was a few days after the last day of the follow-up period (March 31, 2020), excessive mortality attributable to SARS-CoV-2 was unlikely to have impacted the data used for this study.

### Statistical analysis

Values were expressed as the mean ± standard deviation, median, interquartile range, or frequency. Group differences in numeric variables were assessed by analysis of variance or Kruskal–Wallis test, and frequency differences were evaluated with the Chi-squared test. Incidence rates were calculated per 10,000 person-years. Survival curves were calculated using the Kaplan–Meier method, and differences in the survival curves among the quintiles of AAT and hsCRP were assessed using the log-rank test. The Cox proportional hazard model was used to identify factors independently associated with all-cause death. Proportional hazard assumptions were verified using the Schoenfeld residuals test. Statistical analyses were performed using JMP 15.1.0 software (SAS Institute, Cary, NC, USA), while the Cox proportional hazard model analysis and a penalized cubic regression spline model analysis were performed using R 4.0.0 software^31^ with the survival package and the pspline packages, respectively. A *P*-value less than 0.05 was considered to be statistically significant.

## Data Availability

The datasets analyzed during the current study are not publicly available due to ethical reasons but are available from the corresponding author upon reasonable request.
